# Scalp cooling therapy in chemotherapy-induced alopecia: addressing variability in cooling duration and efficacy

**DOI:** 10.1007/s00520-025-10058-y

**Published:** 2025-11-01

**Authors:** Lily Kaufman, Lilia Valentic, Lauren Malley, Christina Icksarus, Lucy Rose, Brittany Dulmage

**Affiliations:** 1 College of Medicine, The Ohio State University, Columbus, OH USA; 2 Department of Dermatology, College of Medicine, The Ohio State University Wexner Medical Center, Columbus, OH USA

**Keywords:** Chemotherapy-induced alopecia, Scalp cooling therapy, Cooling duration, Hair preservation, Cancer supportive care, Infusion center efficiency

## Abstract

**Purpose:**

Chemotherapy-induced alopecia (CIA) is a distressing side effect of cancer treatment with significant psychosocial consequences. Scalp cooling therapy (SCT) reduces the risk of CIA, but variability in pre- and post-infusion cooling durations limits its standardization and broader implementation. This review aims to examine current practices, identify factors contributing to variation, and explore strategies for optimizing SCT protocols.

**Methods:**

We conducted a narrative review of published literature on SCT, including studies evaluating efficacy across chemotherapy regimens, SCT duration, patient factors such as hair type and race, and institutional barriers. We also reviewed studies assessing innovations aimed at reducing chair time and improving feasibility of SCT delivery in diverse clinical settings.

**Results:**

Pre-cooling durations typically range from 5–30 min, while post-cooling times vary from 15 min to 4 h, often without clear evidence for longer durations. Taxane-based regimens show higher hair preservation success compared to anthracyclines. Patient-specific factors (e.g., hair texture, race), staff availability, chair time, and equipment limitations all impact SCT implementation. Portable SCT systems and cooling stations outside the infusion chair have been proposed to improve workflow. Limited data suggest shorter post-cooling may be equally effective and better tolerated.

**Conclusion:**

SCT is an effective intervention for CIA, but wide variation in implementation presents challenges. Standardizing cooling durations based on evidence and patient-centered considerations is essential to improve SCT access, patient satisfaction, and healthcare efficiency. Further research is needed to evaluate shortened protocols, portable systems, and pharmacologic adjuncts that may enhance the feasibility and effectiveness of SCT.

## Introduction

### Background on scalp cooling therapy

Chemotherapy-induced alopecia (CIA) is one of the most distressing side effects for patients undergoing cancer treatment, with substantial psychosocial impacts including decreased self-esteem, altered body image, and impaired quality of life [1, 2]. Scalp cooling therapy (SCT) has emerged as an effective intervention to mitigate CIA, offering patients a means to preserve hair during treatment [3–5]. As SCT becomes increasingly integrated into oncology care, it is important to consider not only its efficacy but also how variability in implementation affects patients and healthcare systems. One challenge is a lack of evidence-based standardized guidance regarding the duration of pre- and post-cooling, which can vary significantly between manufacturer guidelines, treatment regimens, and institutions [6]. These inconsistencies may lead to unnecessarily prolonged treatment sessions, impacting patient satisfaction or inappropriately short sessions, sacrificing clinical efficiency [7] [8]. This paper explores the rationale for SCT cooling durations, the variability in practice, and its implications for patients and health systems.

SCT is a noninvasive method used to reduce the risk of CIA that involves cooling the scalp before, during, and after chemotherapy infusions to protect hair follicles from cytotoxic damage [9, 10]. Scalp hypothermia can be achieved using FDA-approved automatic systems that circulate coolant through flexible caps to maintain a consistent low temperature throughout treatment [6, 11]. Alternatively, patients may use manually applied frozen gel caps, which must be pre-frozen, often with dry ice, stored in a cooler, and changed every 25–30 min by a caregiver during treatment [6, 12]. SCT is most effective with taxane- and anthracycline-based chemotherapy regimens [9, 13]. The primary mechanism relies on vasoconstriction, which reduces blood flow—and thus chemotherapy exposure—to hair follicles [9]. Additionally, SCT may lower the metabolic activity and chemotherapy uptake of follicular cells, further reducing their susceptibility to damage [10].

To maximize SCT effectiveness, patients undergo a period of scalp cooling prior to chemotherapy administration (pre-cooling) and continue cooling after the infusion ends (post-cooling) [6]. However, the optimal duration of these periods is unclear and not standardized, resulting in considerable variation in practice [14–17]. Some institutions adopt fixed times based on manufacturer guidelines, while others adjust durations based on regimen or workflow constraints. This lack of standardization can increase burden on both patients and clinical resources and impact the effectiveness of SCT [6, 8].

We conducted a narrative review of published literature on SCT, including studies evaluating efficacy across chemotherapy regimens, SCT duration, patient factors such as hair type and race, and institutional barriers. We also reviewed studies assessing innovations aimed at reducing chair time and improving feasibility of SCT delivery in diverse clinical settings.

### Implications of longer-than-needed cooling times

Extended pre- and post-cooling durations significantly increase the overall time patients spend at the infusion center, adding hours to an already lengthy, emotionally taxing, and often physically uncomfortable experience [7]. For patients balancing other responsibilities such as work, childcare, and transportation, this added time can create substantial logistical challenges and frustration [18], and may impact willingness to initiate or continue SCT, particularly among patients with limited support systems or long commutes. The added burden may also contribute to treatment fatigue and undermine quality of life, counteracting some of SCTs intended benefits. Patients undergoing SCT have reported headaches and discomfort due to chin strap placement, pressure, and cooling from the cap, leading to frustration with prolonged treatment times [7].

From a systems perspective, prolonged SCT durations place added strain on infusion centers [19]. Extended chair times can reduce patient throughput, contributing to scheduling delays and inefficiencies. Nursing staff are required to monitor patients for longer periods, adding to their workload and potentially limiting their availability for other patient care duties [20]. Equipment such as cooling caps and machines remain occupied for longer stretches, reducing availability for other patients [8, 20].

## Factors contributing to variability in SCT duration and efficacy

### Patient-specific factors

Despite growing use of SCT, variability in patient and institutional factors, combined with a lack of established guidelines, underscores the need to better understand and standardize the determinants of cooling time and efficacy. Chemotherapeutic drugs vary in their cytotoxic effects due to differences in mechanism of action, metabolism, and the interaction with patients' genetic predispositions [14, 21]. SCT employed during taxane-based regimens in has been shown to be more effective in reducing CIA [4, 22]. In a recent retrospective study, 100% of patients receiving paclitaxel-only treatment and 88% of patients receiving docetaxel-based chemotherapy had effective hair preservation with the use of SCT [22]. Another retrospective study found that SCT had favorable hair retention results in over 95% in patients receiving paclitaxel-containing regimens and docetaxel-containing regimens. Anthracycline-based regimens in conjunction with SCT have not shown as effective results in hair retention [4, 14]. Additionally, for regimens containing both anthracyclines and taxanes, the order in which the two are administered has an effect on SCT success, with retrospective studies showing a hair retention rate of 44% in sequential therapy starting with anthracyclines compared to a retention rate of 73% in sequential therapy starting with taxanes [22].

Hair type is another patient-specific factor that can contribute to SCT efficacy. Literature is sparse regarding the success of SCT in varying hair types, indicating a need for further research to better understand its effects on a diverse patient population. A phase II feasibility study analyzed the efficacy of SCT in Black women (*n* = 15) on either non-anthracycline or anthracycline-based regimens, and found that hair loss prevention with SCT was only successful in one patient. The explanation for this discrepancy is unclear, but is it thought that hair thickness, hair volume, and design of the cooling cap could play a role [23]. To improve the rates of SCT success in patients with textured hair, alternative scalp preparation approaches have been proposed, including avoiding the use of hair extensions with synthetic hair, straightening or relaxing hair, and avoiding hairstyles that would leave large portions of the scalp unexposed to SCT [24]. There may also be differences in success rates depending on the type of cap used. A different retrospective study analyzing hair retention in patients using SCT static capping saw that there was no difference in success based on patient race/ethnicity or hair characteristics in patients [4]. This discrepancy in the minimal existing data further highlights the importance of expanding research to involve patients with a variety of hair types.

### Facility and staffing constraints

Facility constraints can also impact the usage and success of SCT. One concern found to be held by healthcare professionals is the limited access and availability of scalp cooling caps, posing a major issue for equity in distribution of the caps [20, 25]. Another major concern found amongst healthcare workers was the feasibility of extending patients’ stays in the treatment room to accommodate for SCT [11, 20, 25]. SCT increases the time before and after each infusion treatment, impacting the total amount of patients that can be seen [11]. Staffing availability and training creates an issue for optimal SCT, as extended cooling times increase the nursing workload [8, 14, 19, 20]. An article reviewing the implementation of a cold cap program at a community health breast center recognized the importance of role delineation in terms of administering SCT. The task became too time consuming for nurses and was a source for potential administration mistakes, and the program therefore began utilizing specifically trained “cold cappers” [26]. These practical challenges, including the time-intensive nature of the procedure and variability in staff capacity and device effectiveness, limit the feasibility of establishing uniform SCT protocols across institutions [27]. Current literature recognizes these constraints of implementing SCT; however, further exploration is required to analyze the impacts of these factors on patients and SCT cooling times.

### System-level and policy factors

Broader policy and structural factors influence the implementation and sustainability of SCT. Insurance reimbursement remains inconsistent, with many payers classifying SCT as a cosmetic or non-essential service, thereby limiting patient access and discouraging institutional investment in equipment [11, 19]. The absence of national or international guidelines specifying standardized pre- and post-cooling durations contributes to variability across centers, as institutions often rely on manufacturer recommendations or internal protocols developed through trial and error [6, 8, 14, 17]. Additionally, the high upfront cost of SCT systems and maintenance contracts may serve as barriers for community or resource-limited oncology centers, further widening disparities in availability [20]. Coordinated policy efforts to establish evidence-based protocols, expand reimbursement, and promote equitable device access could help reduce variability and improve both patient and system-level outcomes.

## Specific scalp cooling times by chemotherapeutic agent

In most studies of SCT, pre-cooling times ranged from five to 30 min prior to the start of intravenous chemotherapy infusion [28–31], with 30 min being the generally accepted standard for pre-cooling time [6]. In contrast, there is no consensus regarding post-infusion cooling time, with post-cooling times ranging from 15 min to four hours [14, 28–32]. Variations in post-cooling times are generally implemented on the basis of chemotherapy drug class and dosing regimen. At many institutions, weekly paclitaxel treatment is followed by 90 min of post-cooling, while longer times are used for other chemotherapy agents such as every-three-week docetaxel, which is followed by three hours of post-cooling [14]. Some studies have asserted that 20 min of post-cooling is sufficient for single-agent taxane chemotherapy, with similar success in preventing CIA relative to 45 min of post-cooling [16–18]. Furthermore, a study comparing the efficacy of 30 min and two hours of post-cooling found no significant difference in hair preservation for patients treated with paclitaxel or epirubicin and cyclophosphamide, concluding that long-duration SCT may result in unnecessary patient discomfort from side effects such as headaches, neck pain, lightheadedness, and nausea [33]. The impact of varying pre- and post-cooling times represents an important area for further research to determine SCT regimens that optimize treatment efficacy while reducing unwanted side effects. Studied post-cooling times are summarized in Table 1. Across studies, post-cooling durations most commonly range from 45–90 min for taxane-based regimens and 90–120 min for anthracycline-containing regimens, although no standardized or evidence-based consensus currently exists [4, 14–18, 22, 23, 28–40].

**Table 1:** SCT times reported in the literature

Study	Drug	Scalp cooling type	Post-infusion cooling time(s)	Results
Taxanes				
van den Hurk et al. (2012) [15]	Docetaxel	Dynamic (Paxman machine)	45 min, 90 min	Shorter cooling time reduced head covering use; 95% of the 45-min group did not require head coverings compared to 79% of the 90-min group (*p* = 0.04)
Komen et al. (2016) [16]	Docetaxel	Dynamic (Paxman machine)	20 min, 45 min	No significant difference in head covering use between groups; 73% of the 20-min group did not require head coverings compared to 79% of the 45-min group (*p* = 0.5)
Lugtenberg et al. (2022) [17]	Paclitaxel	Dynamic (Paxman machine)	20, 45, and 90 min	No significant difference in head covering use between groups (*p* = 0.29); no significant difference in the Dean scale or NCI CTCAE grade (*p* = 0.38) between groups
Lemenager et al. (1997) [28]	Docetaxel	Static (Spenco Hypothermia Cap)	15 min	85.6% (n = 83) success (defined as WHO grade ≤ 2 with no wig use); minimal side effects and no scalp metastases observed during 4–12 month follow-up
Anthracyclines				
Komen et al. (2019) [14]	FEC (5-fluorouracil, epirubicin, cyclophosphamide)	Dynamic (Paxman machine)	90 min, 150 min	Moderate-complete alopecia in 49% of 90-min group and 33% of 150-min group; longer cooling reduced severe alopecia (*p* = 0.02) but not head covering use (*p* = 0.2)
Anderson et al. (1981) [30]	Doxorubicin	Static (gel packs)	30 min	Severe alopecia avoided in 78.6% of patients; 21.4% had severe/total alopecia requiring a wig; 100% efficacy with normal liver function but dropped to 33% with impairment
Dean et al. (1983) [31]	Doxorubicin and cyclophosphamide	Static (ice packs, Kold Kap)	30–40 min	Kold Kap more effective than ice packs; ≤ 50% hair loss in 63% of Kold Kap vs. 56% of ice packs; < 25% hair loss in 51% of Kold Kap vs. 33% of ice packs; ice pack efficacy declined at > 50 mg doxorubicin compared to Kold Kap (*p* = 0.003)
Taxanes and Anthracyclines				
Auvinen et al. (2010) [29]	Doxorubicin, docetaxel, FEC, or combination therapy	Static (cold cap)	15–20 min	Major alopecia avoided in 79.7% overall; grade 2 alopecia in 16.7% (docetaxel), 23.5% (FEC), 18.2% (docetaxel followed by FEC), and 0% (doxorubicin); only 20.3% used a wig
Kang et al. (2024) [18]	Taxane-based, anthracycline-based, or combined chemotherapy regimens	Dynamic (Paxman machine)	20 min after taxane-based therapy; 90 min after doxorubicin-based or combination therapy	PCIA at 6 months in 52% of control group and 13.5% of scalp cooling group (*p* < 0.001); scalp cooling reduced distress (*p* = 0.02) and head covering use (wigs: 32% vs. 17%, *p* = 0.03; scarves/caps: 71% vs. 46%, *p* = 0.005) but not hair density (*p* = 0.53)
Carton et al. (2024) [33]	Epirubicin/cyclophosphamide (EC) followed by paclitaxel	Dynamic (Paxman system)	30 min, 120 min	Longer cooling time had no significant effect on hair loss after EC (*p *= 0.41) or paclitaxel (*p* = 0.67); longer cooling increased discomfort during first session (*p* = 0.039)
Villarreal-Garza et al. (2021) [22]	Paclitaxel v. docetaxel-based v. anthracycline-based	Dynamic (DigniCap system)	60–120 min	Grade 2 alopecia avoided in 100% of paclitaxel-only and 88% of docetaxel groups; 73% success when paclitaxel given before AC vs. 44% when AC given first
Nangia et al. (2017) [34]	Paclitaxel v. docetaxel	Dynamic (Paxman machine)	90 min	Hair preservation in 50.5% of cooling group v. 0% of control group (*p* < 0.001); greater efficacy with taxanes (59%) than anthracyclines (16%)
Bajpai et al. (2020) [35]	Anthracycline followed by taxane	Dynamic (Paxman machine)	90 min	Hair preservation in 56.3% of scalp cooling group vs 0% in control (*p* = 0.00004); scalp cooling significantly increased hair regrowth at 6 weeks (89% vs 12%, *p* < 0.0001) and 12 weeks (100% vs 59%, *p* = 0.0003); more effective when taxane given first (77% vs 33%, *p* = 0.0307)
Macduff et al. (2003) [38]	Epirubicin, docetaxel	Static (cold cap)	45 min	Significantly less hair loss in cold cap group during mid to late treatment (T3-T6; *p* < 0.05); limited protection from alopecia (only 25–31% of cold cap group met success criteria by T4-T6)
Breed et al. (2025) [39]	Adriamycin and cyclophosphamide v. docetaxel v. paclitaxel v. 5-fluorouracil, epirubicin, and cyclophosphamide	Dynamic (primarily Paxman machine, some DigniCap system)	20 min, 45 min, 90 min	Hair preservation comparable across regimens with PICT < 85 min vs ≥ 85 min; longer cooling reduced efficacy for docetaxel 75 mg/m^2^ (*p* = 0.0002)
Weaver et al. (2024) [4]	Paclitaxel-containing v. docetaxel-containing v. doxorubicin-containing v. cisplatin	Static (Penguin Cold Cap)	3–7 h	92.1% retained ≥ 50% hair (median retention 75%); success lower with doxorubicin (71.4%) vs. paclitaxel (95.7%) and docetaxel (96.6%) (*p* = 0.018)
Other				
Dilawari et al. (2021) [23]	Non-anthracycline or anthracycline regimens	Dynamic (Paxman machine)	45 min, 90 min	Low efficacy in Black patients; MDS grade ≥ 3 alopecia in 73% of patients (11/15); only 1/15 (6.7%) achieved hair preservation (≤ 50% loss); scalp cooling increased alopecia (*p* < 0.001) and distress scores (*p* = 0.04); study terminated early due to lack of efficacy
Rugo & Voight (2018) [36]	Various (meta-analysis)	Any type (meta-analysis)	30–60 min	Significant alopecia in 93% of control v. 54% of scalp cooling group; scalp cooling reduced severe alopecia (*p* < 0.00001) and alopecia grade (*p* < 0.0001)
Katsimbri et al. (2000) [37]	Taxanes v. taxanes and anthracyclines v. anthracyclines v. etoposide	Static (Penguin Cold Cap system)	90 min	Hair preservation in 81% overall; 88% in taxane group, 100% in anthracycline and etoposide groups, but only 36% in taxane + anthracycline group (*p* < 0.01)
Brook et al. (2024) [40]	Various (taxanes, anthracyclines, gemcitabine, irinotecan, etc.)	Dynamic (primarily Paxman machine, some DigniCap system)	90 min *varied by drug and hospital protocol	Hair preservation (WHO score 0/1) in 53% of patients; 56% did not wear a head covering at final session; number of scalp cooling sessions strongly predicted success (*p* <.001)

## Future directions

Despite the challenges of implementing SCT, several potential solutions exist. Complementary pharmacologic methods that may increase the efficacy of SCT are under investigation. Topical vasoconstrictors such as ephedrine and naphazoline may reduce blood flow to the scalp prior to chemotherapy administration, limiting drug delivery to hair follicles and mimicking the effects of cooling. These agents may have the potential to offer patients easier administration and prolonged vasoconstriction beyond the infusion period, potentially enhancing the effectiveness of SCT [41]. Animal studies have shown promising results, although further studies including human trials are required to evaluate the efficacy of topical vasoconstrictors for preventing CIA [42].

Early research in CIA prevention explored the efficacy of scalp compression using scalp tourniquets. Controlled trials evaluating the efficacy of elastic or inflated external carotid artery tourniquets demonstrated mixed results. Some studies have found scalp compression to be effective in the prevention of CIA [43–45], while another study determined that scalp compression merely delayed, rather than prevented, CIA [46]. The limited number of studies evaluating scalp compression as a preventative measure for CIA, as well as the fact that existing studies were conducted several decades ago, makes it difficult to draw definitive conclusions about the efficacy of scalp compression in preventing CIA.

In an effort to improve infusion center efficiency and accommodate high-volume chemotherapy schedules, strategies to free up infusion chairs during SCT have become increasingly important. One practical approach is to transition patients from their initial chemotherapy chair to a separate cooling station for the post-infusion cooling period. Additionally, newer technologies such as the Amma Portable Scalp Cooling System offer enhanced portability of SCT, allowing patients to continue their post-chemotherapy cooling phase outside of the infusion center [47]. This model has the potential to reduce chair time and improve patient throughput. While there is a lack of published data on combining dynamic scalp cooling machines with portable systems to fully offload SCT from the infusion setting, the development of such hybrid models or practices in the future could optimize workflow and patient flexibility, warranting further exploration. Looking ahead, future research should also focus on defining evidence-based pre- and post-infusion cooling durations through prospective, regimen-specific studies. Establishing minimum effective cooling times that preserve efficacy while minimizing patient burden and infusion chair occupancy will be critical. Collaborative efforts across institutions could enable the development of standardized SCT protocols that balance clinical outcomes, patient comfort, and operational feasibility.

## Conclusion

SCT offers a promising method to reduce CIA, but variability in cooling times and contributing factors presents significant challenges to its optimization. Differences in chemotherapy regimens, patient characteristics, and institutional policies all have the potential to impact SCT efficacy and feasibility. While a lack of standardization presents clear challenges, existing variability in practice also creates an opportunity to analyze real-world data to better understand how differences in cooling duration affect outcomes. Leveraging such data may help inform future evidence-based recommendations for optimizing SCT protocols. Standardizing pre- and post-cooling durations based on clinical evidence—while accounting for real-world barriers—will be essential to improving patient outcomes, streamlining clinical operations, and promoting equitable access to this supportive therapy.

## Data Availability

No datasets were generated or analysed during the current study.

## References

[1] Lemieux J, Maunsell E, Provencher L (2008) Chemotherapy-induced alopecia and effects on quality of life among women with breast cancer: a literature review. Psychooncology 17(4):317–328. 10.1002/pon.1245 17721909 10.1002/pon.1245

[2] Çelik A, Çinar D, Öztürk Çetin A, Ümit Ünal O (2025) The effect of chemotherapy-induced alopecia on distress and quality of life in male patients with cancer. Oncol Nurs Forum 52(2):126–136. 10.1188/25.ONF.126-136 40028988 10.1188/25.ONF.126-136 PMC12056864

[3] Lambert KA, Albright BB, Anastasio MK, Kaplan SJ, McNally L (2024) Scalp hypothermia to reduce chemotherapy-induced alopecia: a systematic review and meta-analysis. Gynecol Oncol 188:71–80. 10.1016/j.ygyno.2024.06.012 38936283 10.1016/j.ygyno.2024.06.012

[4] Weaver D, Pershing ML, Golden B, Hammel L, Russ PK, Cripe M (2024) Retrospective evaluation of Penguin cold caps for chemotherapy-induced alopecia. Support Care Cancer 32(4):225. 10.1007/s00520-024-08393-7 38472496 10.1007/s00520-024-08393-7 PMC10933155

[5] Contreras Molina M, Álvarez Bueno C, Cavero Redondo I, Lucerón Lucas-Torres MI, Jiménez López E, García Maestro A (2024) Effectiveness of scalp cooling to prevent chemotherapy-induced alopecia in patients undergoing breast cancer treatment: a systematic review and meta-analysis. Cancer Nurs 47(4):319–326. 10.1097/NCC.0000000000001234 37026981 10.1097/NCC.0000000000001234

[6] Rugo HS, van den Hurk C (2024) Alopecia related to systemic cancer therapy. In: Drews RE, Hordinsky M, Vora SR (eds) UpToDate. Wolters Kluwer; Updated August 14, 2024. https://www.uptodate.com/contents/alopecia-related-to-systemic-cancer-therapy?search=scalp%20cooling&source=search_result&selectedTitle=1%7E11&usage_type=default&display_rank=1#H7. Accessed 6 May 2025

[7] Renehan S, Tencic M, Jackson K, Krishnasamy M (2022) Improving preparation for scalp cooling: learning from women undergoing chemotherapy for early-stage breast cancer-the COOL study. J Clin Nurs 31(21–22):3222–3234. 10.1111/jocn.16160 34866261 10.1111/jocn.16160

[8] Ueberroth BE, Kosiorek HE, Nafissi NN et al (2024) Patient and nursing staff perspectives on automated scalp cooling (ASC) for chemotherapy-induced alopecia in breast cancer. Support Care Cancer 32(7):412. 10.1007/s00520-024-08611-2 38842732 10.1007/s00520-024-08611-2

[9] Shin H, Jo SJ, Kim DH, Kwon O, Myung SK (2015) Efficacy of interventions for prevention of chemotherapy-induced alopecia: a systematic review and meta-analysis. Int J Cancer 136(5):E442–E454. 10.1002/ijc.29115 25081068 10.1002/ijc.29115

[10] Bülow J, Friberg L, Gaardsting O, Hansen M (1985) Frontal subcutaneous blood flow, and epi- and subcutaneous temperatures during scalp cooling in normal man. Scand J Clin Lab Invest 45(6):505–508. 10.3109/00365518509155250 4070952 10.3109/00365518509155250

[11] Fischer-Cartlidge E, Ross M, Hernández K, Featherstone A, Haase C (2018) Scalp cooling: implementation of a program at a multisite organization. Clin J Oncol Nurs 22(5):534–541. 10.1188/18.CJON.534-541 30239512 10.1188/18.CJON.534-541

[12] Kruse M, Abraham J (2018) Management of chemotherapy-induced alopecia with scalp cooling. J Oncol Pract 14(3):149–154. 10.1200/JOP.17.00038 29529389 10.1200/JOP.17.00038

[13] Trüeb RM (2010) Chemotherapy-induced hair loss. Skin Therapy Lett 15(7):5–7 20700552

[14] Komen MMC, van den Hurk CJG, Nortier JWR et al (2019) Prolonging the duration of post-infusion scalp cooling in the prevention of anthracycline-induced alopecia: a randomised trial in patients with breast cancer treated with adjuvant chemotherapy. Support Care Cancer 27(5):1919–1925. 10.1007/s00520-018-4432-6 30206728 10.1007/s00520-018-4432-6

[15] van den Hurk CJ, Breed WP, Nortier JW (2012) Short post-infusion scalp cooling time in the prevention of docetaxel-induced alopecia. Support Care Cancer 20(12):3255–3260. 10.1007/s00520-012-1465-0 22539051 10.1007/s00520-012-1465-0

[16] Komen MM, Breed WP, Smorenburg CH et al (2016) Results of 20- versus 45-min post-infusion scalp cooling time in the prevention of docetaxel-induced alopecia. Support Care Cancer 24(6):2735–2741. 10.1007/s00520-016-3084-7 26805558 10.1007/s00520-016-3084-7

[17] Lugtenberg RT, van den Hurk CJG, Smorenburg CH et al (2022) Comparable effectiveness of 45- and 20-min post-infusion scalp cooling time in preventing paclitaxel-induced alopecia - a randomized controlled trial. Support Care Cancer 30(8):6641–6648. 10.1007/s00520-022-07090-7 35501515 10.1007/s00520-022-07090-7 PMC9213299

[18] Kang D, Cho J, Zhao D et al (2024) Scalp cooling in preventing persistent chemotherapy-induced alopecia: a randomized controlled trial. J Clin Oncol 42(26):3115–3122. 10.1200/JCO.23.02374 38843479 10.1200/JCO.23.02374

[19] Novice M, Novice T, Henry NL et al (2022) Identifying barriers and facilitators to Scalp Cooling Therapy through a national survey of the awareness, practice patterns, and attitudes of oncologists. JCO Oncol Pract 18(2):e225–e234. 10.1200/OP.21.00273 34529505 10.1200/OP.21.00273

[20] Shaw JM, O’Brien J, Chua S et al (2018) Barriers and enablers to implementing scalp cooling in Australia: a qualitative study of health professionals’ attitudes to and experience with scalp cooling. Support Care Cancer 26(1):305–312. 10.1007/s00520-017-3849- 28852873 10.1007/s00520-017-3849-7

[21] Shah VV, Wikramanayake TC, DelCanto GM et al (2018) Scalp hypothermia as a preventative measure for chemotherapy-induced alopecia: a review of controlled clinical trials. J Eur Acad Dermatol Venereol 32(5):720–734. 10.1111/jdv.14612 28976026 10.1111/jdv.14612 PMC8127610

[22] Villarreal-Garza C, Mesa-Chavez F, Garza-Ledezma MRA et al (2021) Impact of chemotherapy regimen and sequence on the effectiveness of scalp cooling for alopecia prevention. Breast Cancer Res Treat 185(2):453–458. 10.1007/s10549-020-05968-w 33125621 10.1007/s10549-020-05968-w

[23] Dilawari A, Gallagher C, Alintah P et al (2021) Does Scalp Cooling Have the Same Efficacy in Black Patients Receiving Chemotherapy for Breast Cancer? Oncologist 26(4):292-e548. 10.1002/onco.13690 33512741 10.1002/onco.13690 PMC8018328

[24] Araoye EF, Stearns V, Aguh C (2020) Considerations for the use of scalp cooling devices in Black patients. J Clin Oncol 38(30):3575–3576. 10.1200/JCO.20.02130 32897824 10.1200/JCO.20.02130 PMC7571797

[25] Delgado Rodríguez J, Ramos-García V, Infante-Ventura D et al (2023) Ethical, legal, organizational and social issues related to the use of scalp cooling for the prevention of chemotherapy-induced alopecia: a systematic review. Health Expect 26(2):567–578. 10.1111/hex.13679 36585793 10.1111/hex.13679 PMC10010082

[26] Heery M, Cohen S, Mena Z (2019) Scalp Cooling: Implementing a Cold Cap Program at a Community Breast Health Center. Clin J Oncol Nurs 23(3):237–241. 10.1188/19.CJON.237-241 31099802 10.1188/19.CJON.237-241

[27] Batchelor D (2001) Hair and cancer chemotherapy: consequences and nursing care–a literature study. Eur J Cancer Care (Engl) 10(3):147–163. 10.1046/j.1365-2354.2001.00272.x 11829374 10.1046/j.1365-2354.2001.00272.x

[28] Lemenager M, Lecomte S, Bonneterre ME, Bessa E, Dauba J, Bonneterre J (1997) Effectiveness of cold cap in the prevention of docetaxel-induced alopecia. Eur J Cancer 33(2):297–300. 10.1016/s0959-8049(96)00374-7 9135504 10.1016/s0959-8049(96)00374-7

[29] Auvinen PK, Mähönen UA, Soininen KM et al (2010) The effectiveness of a scalp cooling cap in preventing chemotherapy-induced alopecia. Tumori 96(2):271–275. 10.1177/030089161009600214 20572585 10.1177/030089161009600214

[30] Anderson JE, Hunt JM, Smith IE (1981) Prevention of doxorubicin-induced alopecia by scalp cooling in patients with advanced breast cancer. Br Med J (Clin Res Ed) 282(6262):423–424. 10.1136/bmj.282.6262.423 6780057 10.1136/bmj.282.6262.423 PMC1504234

[31] Dean JC, Griffith KS, Cetas TC, Mackel CL, Jones SE, Salmon SE (1983) Scalp hypothermia: a comparison of ice packs and the Kold Kap in the prevention of doxorubicin-induced alopecia. J Clin Oncol 1(1):33–37. 10.1200/JCO.1983.1.1.33 6668482 10.1200/JCO.1983.1.1.33

[32] Tollenaar RA, Liefers GJ, Repelaer van Driel OJ, van de Velde CJ (1994) Scalp cooling has no place in the prevention of alopecia in adjuvant chemotherapy for breast cancer. Eur J Cancer 30A(10):1448–1453. 10.1016/0959-8049(94)00280-i 10.1016/0959-8049(94)00280-i 7833100

[33] Carton E, Blas AM, Perret C, Le Bihan M (2024) Effectiveness of increasing the scalp cooling duration to prevent alopecia during adjuvant chemotherapy for breast cancer: a randomized pilot study. Support Care Cancer 32(7):410. 10.1007/s00520-024-08579-z 38839667 10.1007/s00520-024-08579-z PMC11153286

[34] Nangia J, Wang T, Osborne C et al (2017) Effect of a scalp cooling device on alopecia in women undergoing chemotherapy for breast cancer: the SCALP randomized clinical trial. JAMA 317(6):596–605. 10.1001/jama.2016.20939 28196254 10.1001/jama.2016.20939

[35] Bajpai J, Kagwade S, Chandrasekharan A et al (2020) Randomised controlled trial of scalp cooling for the prevention of chemotherapy induced alopecia. Breast 49:187–193. 10.1016/j.breast.2019.12.004 31865282 10.1016/j.breast.2019.12.004 PMC7375683

[36] Rugo HS, Voigt J (2018) Scalp hypothermia for preventing alopecia during chemotherapy. A systematic review and meta-analysis of randomized controlled trials. Clin Breast Cancer 18(1):19–28. 10.1016/j.clbc.2017.07.012 28939291 10.1016/j.clbc.2017.07.012

[37] Katsimbri P, Bamias A, Pavlidis N (2000) Prevention of chemotherapy-induced alopecia using an effective scalp cooling system. Eur J Cancer 36(6):766–771. 10.1016/s0959-8049(00)00012-5 10762750 10.1016/s0959-8049(00)00012-5

[38] Macduff C, Mackenzie T, Hutcheon A, Melville L, Archibald H (2003) The effectiveness of scalp cooling in preventing alopecia for patients receiving epirubicin and docetaxel. Eur J Cancer Care (Engl) 12(2):154–161. 10.1046/j.1365-2354.2003.00382.x 12787013 10.1046/j.1365-2354.2003.00382.x

[39] Breed FFI, Breed WPM, Dercksen MW, Ezendam N, Bijlsma MJ, van den Hurk CJG (2025) Shorter post-infusion cooling times at least as effective as longer cooling times - an observational study of taxane- and anthracycline-based chemotherapies in the Dutch Scalp Cooling Registry. Support Care Cancer 33(6):503. 10.1007/s00520-025-09526-2 40434428 10.1007/s00520-025-09526-2

[40] Brook TS, Seetsen T, Dercksen MW et al (2024) Results of the Dutch scalp cooling registry in 7424 patients: analysis of determinants for scalp cooling efficacy. Oncologist 29(10):e1386–e1395. 10.1093/oncolo/oyae116 38869252 10.1093/oncolo/oyae116 PMC11449096

[41] Rossi A, Fortuna MC, Caro G et al (2017) Chemotherapy-induced alopecia management: clinical experience and practical advice. J Cosmet Dermatol 16(4):537–541. 10.1111/jocd.12308 28150447 10.1111/jocd.12308 PMC5540831

[42] Soref CM, Fahl WE (2015) A new strategy to prevent chemotherapy and radiotherapy-induced alopecia using topically applied vasoconstrictor. Int J Cancer 136(1):195–203. 10.1002/ijc.28961 24811525 10.1002/ijc.28961 PMC4342350

[43] Lovejoy NC (1979) Preventing hair loss during adriamycin therapy. Cancer Nurs 2(2):117–121 255102

[44] Li Z, Liu J, Zhuang X et al (1995) Clinical observation of the effects of hair band to alleviate theadriamycin and cyclophosphamide induced hair loss [in Chinese]. Pract Nurs 12:32–33

[45] Pesce A, Cassuto JP, Joyner MV, DuJardin P, Audoly P (1978) Scalp tourniquet in the prevention of chemotherapy-induced alopecia. N Engl J Med 298(21):1204–1205. 10.1056/nejm197805252982121 651958 10.1056/nejm197805252982121

[46] (1978) Scalp tourniquet in cancer therapy. N Engl J Med 299(11):605-606. 10.1056/NEJM197809142991115 10.1056/NEJM197809142991115 683231

[47] (2025) Cooler heads care inc. study to assess the ability of the portable scalp cooling system (PSCS) to prevent hair loss in women receiving chemotherapy for stage I-III breast cancer. ClinicalTrials.gov. NCT05484973. Accessed 11 June 2025

